# Egg survival is reduced by grave-soil microbes in the carrion beetle, *Nicrophorus vespilloides*

**DOI:** 10.1186/s12862-014-0208-x

**Published:** 2014-09-27

**Authors:** Chris G C Jacobs, Yin Wang, Heiko Vogel, Andreas Vilcinskas, Maurijn van der Zee, Daniel E Rozen

**Affiliations:** Institute of Biology Leiden, Leiden University, Sylviusweg 72, 2333 Leiden, BE the Netherlands; Department of Entomology, Max Planck Institute for Chemical Ecology, Hans-Knöll-Straße 8, D-07745 Jena, Germany; Justus-Liebig University, Heinrich-Buff-Ring 26-32, D-35392 Giessen, Germany

**Keywords:** Trade-off, Burying beetle, Egg immunity, Developmental speed

## Abstract

**Background:**

*Nicrophorus vespilloides* eggs are deposited into the soil in close proximity to the decomposing vertebrate carcasses that these insects use as an obligate resource to rear their offspring. Eggs in this environment potentially face significant risks from the bacteria that proliferate in the grave-soil environment following nutrient influx from the decomposing carcass. Our aims in this paper are twofold: first, to examine the fitness effects of grave-soil bacteria to eggs, and second, to quantify egg immunocompetence as a defence against these bacteria.

**Results:**

Our results provide strong evidence that grave-soil microbes significantly reduce the survival of *Nicrophorus* eggs. Females provided with microbe rich carcasses to rear broods laid fewer eggs that were less likely to hatch than females given uncontaminated carcasses. Furthermore, we show that egg hatch success is significantly reduced by bacterial exposure. Using a split-brood design, which controlled for intrinsic differences in eggs produced by different females, we found that eggs washed free of surface-associated bacteria show increased survival compared to unwashed eggs. By contrast, eggs exposed to the entomopathogen *Serratia marcescens* show decreased survival compared to unexposed eggs. We next tested the immune competence of eggs under challenge from bacterial infection, and found that eggs lacked endogenous production of antimicrobial peptides, despite well-developed responses in larvae. Finally, we found that despite lacking immunity, *N. vespilloides* eggs produce an extraembryonic serosa, indicating that the serosa has lost its immune inducing capacity in this species.

**Conclusions:**

The dependency on ephemeral resources might strongly select for fast developing animals. Our results suggest that *Nicrophorus* carrion beetles, and other species developing on ephemeral resources, face a fundamental trade-off between egg immunity and development time.

**Electronic supplementary material:**

The online version of this article (doi:10.1186/s12862-014-0208-x) contains supplementary material, which is available to authorized users.

## Background

Exposure to harmful microbes poses numerous and diverse threats to developing animals [1]. For animals with internal development, microbial pathogens that can directly harm the embryo can be controlled by the surveillance of maternal adaptive and innate immunity [2,3]. By contrast, microbial defence in animals that develop externally is provided by barrier protection from the egg surface, from maternally provided antimicrobials or through intrinsic immunity coordinated by the developing embryo [4]. These modes of protection have been extensively examined in vertebrates [5]. For example, avian egg shells provide direct physical protection against external microbial challenge, while mothers provision eggs prior to laying with a suite of general and specific antimicrobials, such as lysozyme, avidin and ovotransferrin [6], which provide crucial protection to the embryo prior to the maturation of the embryonic immune response. In invertebrates, parents can similarly invest in offspring defence via trans-generational immunity that provides diverse defences against pathogens and parasites that parents have encountered and which may pose specific threats to offspring [7]. This can occur via deposition of antimicrobials onto the insect egg surface, or maternal provisioning of antimicrobials into the egg itself [8-13]. In addition, embryos in some invertebrate species can also mount endogenous defences against pathogen challenge by producing antimicrobial peptides within eggs [9,14,15]. However, this response is not universal and is notably absent in the well-studied model species *Drosophila* [15].

Here we examine the role of egg immunity in the burying beetle *Nicrophorus vespilloides*. This species is particularly suited for this investigation because eggs of this species face considerable challenge from the bacteria they encounter during development [16]. *Nicrophorus* species reproduce on small vertebrate cadavers which they bury in the soil after they are located through volatiles emitted from the carcass. Burying beetle eggs are laid into the soil adjacent to vertebrate carcasses [17]. After a two-three day incubation, eggs hatch and larvae migrate to the carcass where they are communally reared by one or both parents [18]. Caring parents regurgitate food to their developing larvae and also provide protection against insect competitors and predators [17,19]. In addition, parents protect offspring against bacterial competitors growing on the decomposing carcass by depositing antimicrobial secretions, e.g. lysozyme, on the carcass surface [16,20-23]. Parental lysozyme secretion peaks during brood rearing and significantly increases larval survival [20]. Larvae also contribute to brood social immunity by secreting antimicrobials that inhibit bacterial growth [24,25]. They also show a progressive increase in humoral and cellular immunity through development [26]. Although different life stages of the burying beetle show both behavioural and immunological responses to reduce the negative effects of microbial challenge, studies of these responses to date have focussed on post-hatch behaviours and reductions in fitness [16,27,28]. However, pre-hatch reductions in fitness as a consequence of microbial exposure have not been studied; therefore, it remains unknown how or if eggs respond to the adverse environment in which they are laid.

In this study we investigated both the impact of soil-borne bacteria on egg development and the ability of the eggs to mount immune responses. We first measured the consequences of microbial challenge on pre-hatch fitness by assessing egg survival across contrasting environmental conditions. Next, we tested whether antimicrobial peptide genes are expressed in burying beetle eggs in response to infection [29]. Briefly, we show that eggs are significantly harmed by exposure to microbes in grave soil and that eggs lack endogenous immunity. We discuss this lack of an immune response in the light of a trade-off with developmental speed.

## Methods

### General procedures

Experimental animals were taken from an outbred laboratory population derived from wild-caught *N. vespilloides* individuals trapped in Warmond near Leiden in The Netherlands, between May and June 2013. Beetles were maintained in the laboratory at 20°C with a 15:9 hour light:dark cycle. All adults were fed fresh chicken liver twice weekly. To collect eggs, non-sibling pairs of beetles were allowed to mate for 24 hours, after which the female was removed and provided with either a Fresh or Aged mouse carcass weighing 24-26 g in a 15 cm × 10 cm plastic box filled with approximately 1–2 cm of soil. The state of found carcasses in the field across the breeding season remains unclear. Accordingly our treatments are meant to represent different extremes of the potential continuum of carcass decay. Following [16], Fresh carcasses are defined as mice that were thawed after removal from the freezer and provided directly to mated females, while Aged carcasses were allowed to age for 7 days on top of commercial peat soil before mated females were added.

### Egg survival

Mated females were provided with either a Fresh (n = 35) or Aged (n = 35) carcass in order to quantify the role of carcass age on egg number and survival. Commencing the morning following set-up, boxes with mice and females were visually inspected every 12 hours to determine the timing of egg appearance. 48 hours later, eggs were removed from the soil and allowed to hatch in petri plates at 20°C containing 1.5% water agar. Egg hatch was monitored every 3 hours until no further hatching was observed.

To examine the role of soil-borne microbes on egg hatch we carried out two different experiments using a split-brood design. In the first experiment, eggs were collected from the soil from females provided with a Fresh carcass (n = 32). Each brood with a minimum of 20 total eggs (n = 30) was split into two treatment groups. Half of each brood was gently rinsed in sterile water and then allowed to hatch on sterile 1% water agar. The other half of each brood was rinsed in a solution containing the entomopathogenic bacterium *Serratia marcescens* at a density of 10^8^/ml, after which eggs were placed to hatch onto sterile water agar. The split-brood design allowed us to control for intrinsic differences in the hatch rate of broods from different females.

In the second experiment, eggs were collected from females provided with an Aged carcass. Using a split-brood design and with the same minimum threshold for inclusion of 20 eggs (n = 29), broods were divided into two treatment groups. A control group of washed eggs from each family was transferred to sterile water agar. The other half of each brood was first surface sterilized in an antimicrobial solution of hen egg-white lysozyme (1 mg/ml), streptomycin (500 μg/ml) and ampicillin (100 μg/ml), and then placed onto water agar plates to hatch. Previous experiments have shown that eggs thus treated are free of bacteria [24].

To assess the ability of *N. vespilloides* eggs to withstand desiccation we collected eggs from soil 15 hours after females were given a carcass. This cut-off was used to ensure that eggs were roughly of the same age. Eggs were placed onto 1% sterile water agar plates and incubated for 24 hours at 20°C. Next, eggs were transferred to glass petri dishes and allowed to hatch at 20°C with either 75% or 90% relative humidity (RH) in an environmental chamber. A separate set of eggs was retained on water agar as a control. The proportion of hatched eggs was scored after 3 days.

### Experimental infection of *N. vespilloides* eggs and larva

To examine the capacity for eggs to mount an immune response against microbial challenge, eggs were experimentally infected with a concentrated solution of *Escherichia coli* and *Micrococcus luteus*. Eggs were collected 15 hours after females were provided with a fresh carcass and then kept at 20°C for 24 hours on 1% sterile water agar. Next, eggs were pricked with a sterile 1 micron tip tungsten needle (Fine Science Tools) dipped into bacterial solution (septic injury) or with a sterile needle alone (sterile injury). After infection/sterile injury, eggs were incubated for 6 hours at 20°C before RNA extraction. For larval infection we allowed eggs to hatch on 1% water agar. Larvae between 0–24 h old were then pricked with either a sterile needle or, with a needle previously dipped into the same bacterial solution as above. Larvae were incubated for 6 hours at 20°C before RNA extraction.

### RNA extraction and real-time quantitative RT-PCR (qRT-PCR)

Total RNA of 5–10 eggs or larvae was extracted using TRIzol (Invitrogen) after which the RNA was purified and DNA digested on column with the RNeasy kit (Qiagen). The quality of the RNA preparation was confirmed spectrophotometrically. One microgram of total RNA was used for cDNA synthesis. First strand cDNA was made using the Cloned AMV First Strand Synthesis kit (Invitrogen). Each qRT-PCR mixture (25 μl) contained 2.5 ng of cDNA, and the real-time detection and analyses were done using SYBR green dye chemistry with the qPCR kit for SYBR Green I (Eurogentec) and a CFX96 thermocycler (Biorad). Thermal cycling conditions used were 50°C for 2 min, 95°C for 10 min, then 50 cycles of 95°C for 15 s, 60°C for 30s, 72°C for 30s. This was followed by dissociation analysis of a ramp from 65 to 95°C with a read every 0.5°C. Relative quantification for each mRNA was done using the Livak-method [30]. The values obtained for each mRNA were normalized by RPL7 mRNA amount. Total RNA for each treatment was isolated twice (biological replication) and each sample was measured by qRT-PCR twice (technical replication). Comparisons between treatments (untreated, sterile injury and septic injury) were performed within one brood.

### Immune-related genes and primers used for qRT-PCR

Real-time PCR oligonucleotide primers were designed using Primer3Plus (http://primer3plus.com/cgi-bin/dev/primer3plus.cgi) by applying the rules of highest maximum efficiency and sensitivity to avoid the formation of dimers, hairpins and other artefacts. The following immune-related genes were examined: *Attacin 2*, *Defensin 1*, *Defensin 2*, *Coleoptericin 1*, *Coleoptericin 2*, *Coleoptericin 3* and the normalizer of qRT-PCR *ribosomal protein 7* (*RPL7*). Sequences of immune-related genes were derived from [29], and primer pairs of respective target genes were designed for qRT-PCR (Table 1).

**Table 1:** **Primers for immune sequences of** ***Nicrophorus vespilloides***

**Gene**	**Forward primer**	**Reverse primer**
*Attacin2*	5’-ACGTCACAGGAGAAGAGCTGA-3’	5’-TCGGAAGGCCTGTGTGTGTA-3’
*Defensin1*	5’-GTCGATACGCCCATCGGTTC-3’	5’-GCAATTGCAGACTCCGTCGA-3’
*Defensin2*	5’-AGAGGTGCATGCGATCTGTT-3’	5’-TGTGCCTTTGGTGTATCCGT-3’
*Coleoptericin1*	5’-CGAAACGGTGGTGAACAGGT-3’	5’-TGCATTGGTTGTACCGTCGG-3’
*Coleoptericin2*	5’-TGGTCTCCGCCGAATCCTAA-3’	5’-GCACCTGGTCTTTCGTGCTT-3’
*Coleoptericin3*	5’-ACTTTGGCGCGAGTCGATTT-3’	5’-TTGATCGCCCAACTCGCTTC-3’
*RPL7*	5’-TGCCATCAAGAAGCGCTCTG-3’	5’-GCGCTCTTGGCTTGATGGAT-3’

### Embryo fixation and microscopy

The extraembryonic serosa in *Tribolium castaneum* is known to be involved in both desiccation resistance [31] and endogenous immune competence of the eggs [15]. All insect species studied to date, with the exception of one group of higher flies [32,33], develop a serosa [34]. Embryonic development of *N. vespilloides* however, has not been studied. To examine the development of the serosa in *N. vespilloides,* fixed eggs were visualized under the confocal microscope (5× magnification). Eggs were placed onto 1% water agar plates at 20°C and left for 24 hours to ensure that enough time had passed to develop the serosa. Next, eggs were fixed for 18 hours at room temperature in a solution of 4 ml phosphate buffered saline (PBS), 1 ml 37% formaldehyde and 5 ml of heptane. They were removed from the fixative and cut in half with a scalpel. The cut eggs were washed 3 times in PBS-Tween and then stained with DAPI for 2 hours at room temperature. After staining, the eggs were washed 3 times with PBS-T and embedded in glycerol on a glass bottom petri dish. Samples were studied with a Zeiss Cell Observer.

## Results

### Egg number and survival is reduced in the presence of an Aged carcass

**Figure 1:**
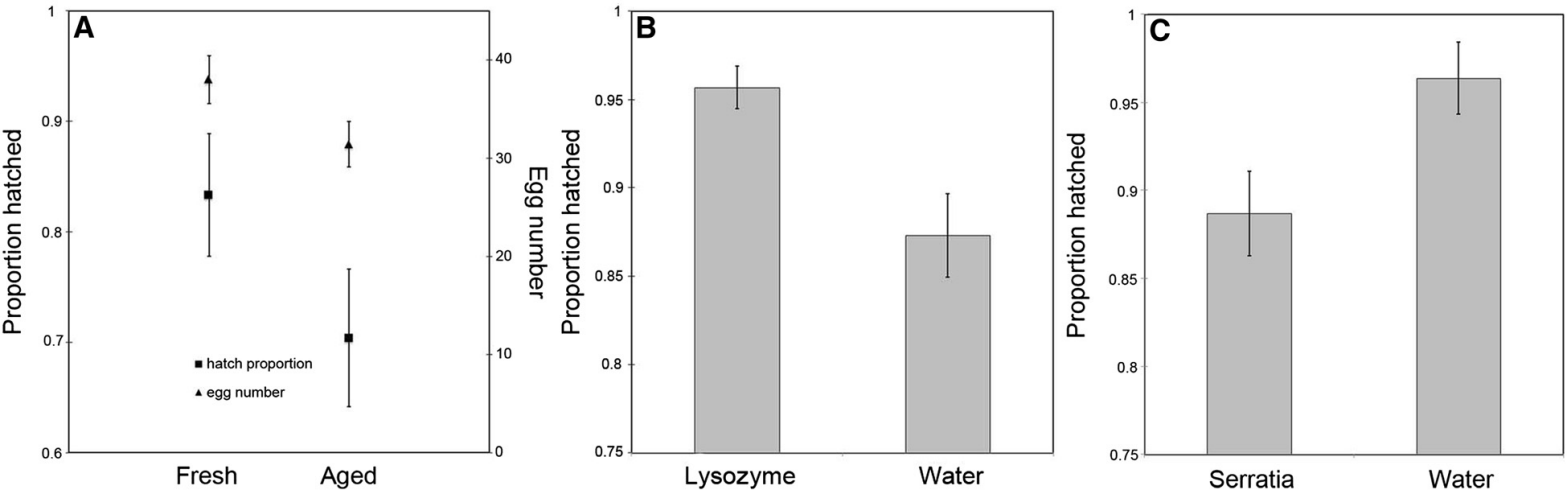
**Egg survival and number under different treatments. A)** Both egg number and egg survival are significantly lower when in the presence of an Aged carcass. **B)** Eggs collected from an Aged carcass show increased survival when sterilized, indicating the negative effect of high bacterial numbers surrounding Aged carcasses. **C)** Eggs collected from a Fresh carcass show decreased survival when experimentally exposed to the entomopathogenic bacterium *S. marcescens*.

Females that were provided with an Aged carcass laid significantly fewer eggs than females that were provided with a Fresh carcass (two-tailed Mann–Whitney U Test, P = 0.012, Figure 1A). In addition, the survival of eggs laid by females provided with an Aged carcass was significantly lower than the survival of eggs laid near a Fresh carcass (two-tailed Mann–Whitney U Test, P = 0.011, Figure 1A). Combining these to obtain an overall estimate of brood size, by taking the product of egg number and hatch proportion, we find that broods laid near to Fresh carcasses are significantly larger than those laid near to Old carcasses (Fresh: 32.57 ± 3.01 vs Old: 23.11 ± 2.61; two-tailed Mann–Whitney U Test, P = 0.005). Together these data show that pre-hatch fitness is reduced by the presence of an Aged carcass.

To test the idea that bacteria in the soil cause this reduction in survival, we split broods laid near an Aged carcass and surface-sterilized one half with an antimicrobial solution while leaving the other half unsterilized. As predicted, if bacteria on the surface of eggs contributed to the failure of eggs to hatch, sterilizing eggs significantly increased egg survival when compared to washing eggs with water (paired t-test, df = 29, p < 0.001, Figure 1B). To further examine the idea that exposure to high bacterial numbers decreases pre-hatch fitness, we again used a split-brood design and experimentally exposed eggs laid near a Fresh carcass to the soil borne entomopathogen *S. marcescens* and compared these to eggs washed in water. Exposure to *S. marcescens* had a pronounced negative effect on pre-hatch fitness (paired t-test, df = 28, p < 0.001, Figure 1C). Notably, the reduction in survival following experimental infection, and the increase in survival following surface sterilization are roughly equivalent. Furthermore, these differences are similar to the differences first observed in untreated eggs laid near Aged and Fresh carcasses. Together, these data strongly indicate that harmful bacteria in the environment of Aged carcasses significantly reduce pre-hatch fitness.

### Antimicrobial peptide expression in response to infection

**Figure 2:**
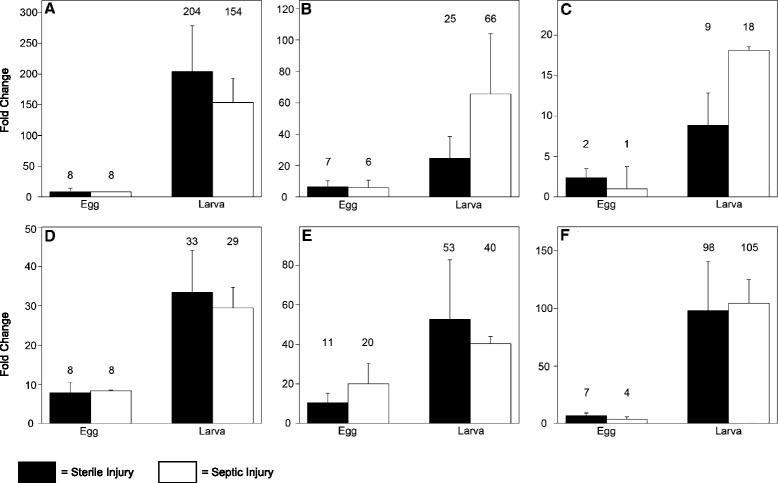
**Expression of antimicrobial peptide genes in response to sterile injury (black bars) and septic injury (white bars).** Whereas larvae show clear induction of all genes tested, eggs show hardly any induction of antimicrobial peptides at all. **A)** *Attacin 2* **B)** *Defensin1* **C)** *Defensin 2* **D)** *Coleoptericin 1* **E)** *Coleoptericin 2* **F)** *Coleoptericin 3.*

Although survival of *N. vespilloides* eggs is reduced in the presence of an Aged carcass, overall egg viability is still quite high; approximately 70% of the eggs still survive even under these challenging conditions (Figure 1A). As we have previously shown that the eggs of *Tribolium castaneum* can induce antimicrobial peptide genes upon infection [15], induction of antimicrobial peptides might also increase survival in adverse conditions for the eggs of *N. vespilloides*. We measured gene expression of several antimicrobial peptides after both sterile injury and septic injury in *N. vespilloides* eggs and larva. Surprisingly, in eggs we found marginal, if any, upregulation of antimicrobial peptide genes after infection (Figure 2). Only one gene (*coleoptericin 2*, Figure 2E) was induced over 10 fold after infection. By contrast, freshly emerged larvae show clear induction of all antimicrobial peptide genes tested (Figure 2). To verify that mRNA levels are lower in the eggs, we compared infected eggs with infected larvae. As expected, transcript levels are higher in larvae (Additional file 1: Figure S1). These data show that although freshly emerged larvae can induce immune genes upon infection, eggs of *N. vespilloides* show very limited AMP inducing capacities.

### Eggs develop an extraembryonic serosa

**Figure 3:**
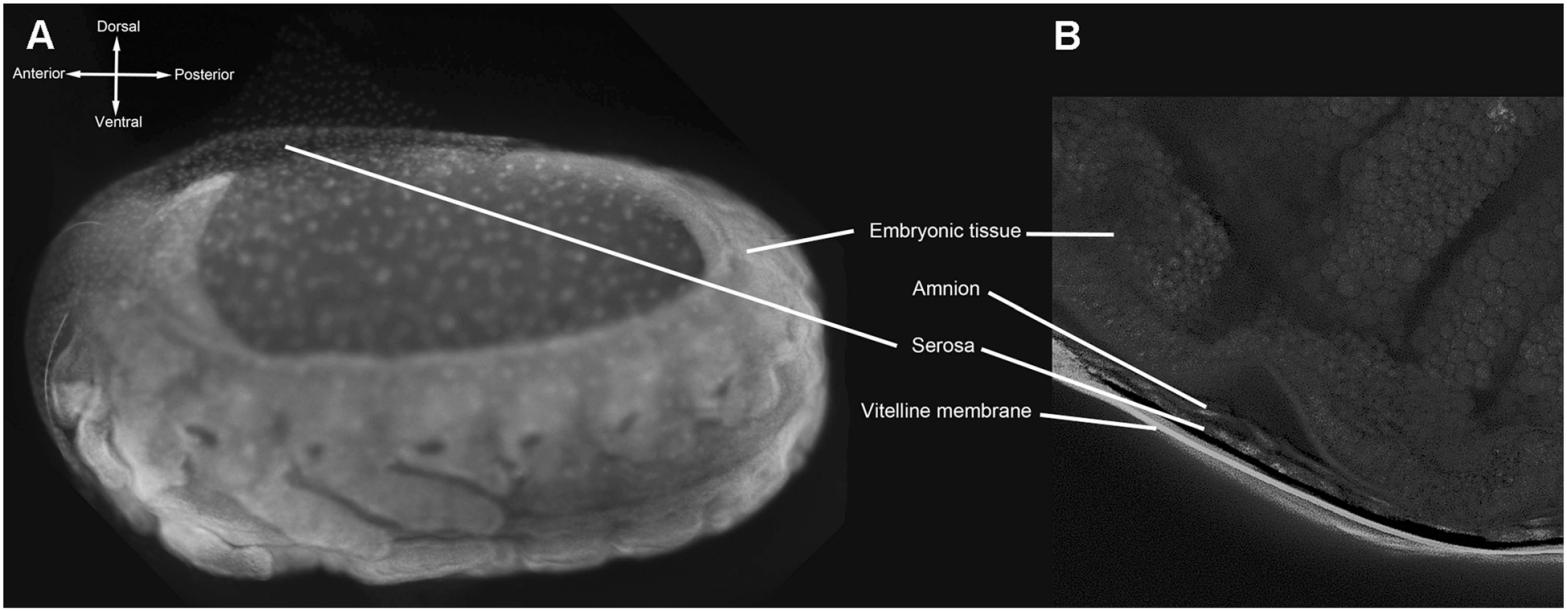
**The eggs of** ***N. vespilloides*** **develop an extraembryonic serosa. A)** Overview of a complete embryo, the developing head is visible at the anterior. The serosal epithelium can be clearly seen just above the head. **B)** Optical section of an *N. vespilloides* egg. The embryo, serosa, amnion and vitelline membrane can be clearly distinguished.

The immune response of *Tribolium castaneum* eggs depends on the presence of an extraembryonic epithelium called the serosa [15]. By contrast, the immune response is poor in eggs of the fruit fly *Drosophila melanogaster* which lack this epithelium. Given the apparent absence of endogenous egg immunity in *N. vespilloides*, we hypothesized that this species, like *Drosophila*, would lack a serosal epithelium. We tested this idea in two ways, first by measuring desiccation tolerance of eggs, as the serosa imparts drought resistance in *T. castaneum* [31], and second by directly examining DAPI stained eggs via confocal microscopy. *N. vespilloides* eggs are highly susceptible to desiccation; egg survival dropped from 92% at 90% RH to 0% at 75% RH (chi-square test, p < 0.001). Although this result, together with the absence of endogenous immunity is consistent with the absence of a serosal epithelium, DAPI-stained confocal microscopy clearly revealed an epithelium around the egg (Figure 3A). This epithelium could easily be distinguished from the amnion in optical sections (Figure 3B) and was identified as serosa.

## Discussion

*Nicrophorus* eggs are deposited into the soil in close proximity to vertebrate carcasses [17]. Eggs in this grave-soil environment are exposed to increased nutrient fluxes from carcass decay that increases the biomass of endogenous bacteria and of bacteria that migrate to the soil from the perforated carcass [35]. Several previous studies have documented the diverse and persistent negative effects of this flora on the survival and growth of developing larvae [16,27,28,36]. Here we extend these findings by showing that carcass associated bacteria also significantly reduce the survival of *Nicrophorus* eggs. We found that females provided with an Aged carcass laid fewer eggs that were less likely to hatch than their female counterparts provided with a Fresh carcass (Figure 1A). In addition, we show that egg hatching success is a direct function of bacterial exposure; eggs washed free of surface-associated bacteria show increased survival compared to unwashed eggs (Figure 1B) while eggs washed in a bacterial solution show decreased survival compared to unexposed eggs (Figure 1C). The overall consequence of this exposure is an approximate 30% decline in potential brood size. This cost, in addition to those already identified at later stages of beetle development, clarify the risks to *Nicrophorus* of rearing young on microbe-rich contaminated carcasses.

The carcasses that *Nicrophorus* larvae rely upon are classical bonanza resources that are unpredictable in time and space. Parents modify the carcass in numerous ways that increase larval growth and survival. The carcass is buried, stripped of fur and coated with both antibacterial and antifungal compounds [20,22,23,27,37]. In addition, parents defend the carcass before and following the arrival of larvae from insect competitors like flies or other carrion beetles [16,17,38]. In contrast to these elaborate behaviours used to defend larvae, there is surprisingly little direct evidence for parental defence of eggs. Earlier research failed to find any lysozyme-like activity inside or on the *N. vespilloides* egg [24], suggesting an absence of direct antimicrobial provisioning. And although antiseptic volatiles secreted by parents into the soil surrounding the carcass may provide an indirect benefit to eggs, this is as yet untested [37].

Why is egg defence apparently missing in this species? One possible explanation is that explicit care of eggs trades-off with carcass maintenance and defence. Thus rather than investing in individual eggs, parents instead invest in preserving the resource that will provide an aggregate benefit to any larvae that survive the egg-stage and eventually migrate to the carcass. Consistent with this idea, egg production in *Nicrophorus* does not appear to engender significant costs [39], the number of eggs observed in experimental *Nicrophorus* broods typically exceeds the number of larvae found on the carcass and infanticidal culling is common [40,41]. It is likely that there is further mortality in the field where eggs face additional predation risks that are not present in the lab. Finally, by secreting antimicrobials on the carcass surface, parents can maintain the carcass in a suitable state for extended time periods, assuming it is found prior to significant decomposition. Also, because parents prefer a Fresh over an Aged carcass [16], eggs may not have been selected to be able to cope with high levels of associated bacteria on extensively decomposed carrion.

A second possibility is that explicit defence is prohibitively expensive, especially when, even in its absence, egg survival is quite high (Figure 1C). This contrasts markedly with other species, like earwigs, where untended eggs challenged with mold infection show far more dramatic declines in hatch success [42]. Although we do not know the cause for high rates of intrinsic survival, it is possible that this is facilitated by the barrier defence provided by the extraembryonic serosa (Figure 3). If so, this would be consistent with an immune-related function for the *Nicrophorus* serosa, even if the serosa in this species appears not to extensively regulate endogenous AMP production as it does for eggs of *Tribolium castaneum*. A challenge for future studies is to explicitly test this hypothesis using RNAi based targeted knock-downs of the developmental genes that regulate the production of this extraembryonic tissue.

Even in the absence of parental protection, eggs of some insects retain the capacity to generate an endogenous immune response against pathogen challenge [9,14,15]; this is thought to be one important cause for the low incidence of parental care in insects [43,44]. Yet this endogenous response is absent in *N. vespilloides*. In that respect, there are striking similarities in development beween *N. vespilloides* and *D. melanogaster*. Both species lack inducible egg immunity and develop on ephemeral resources that favour rapid development times [15,17,45], and specifically rapid embryonic development. Embryonic development in *Nicrophorus* is approximately 3–6 times faster than *Tribolium* and *Manduca* [46,47], and about 20 hours faster than *Aedes*, which are known to go into diapause, meaning they have to survive for a long time until the conditions favour hatching [48]. By contrast, *Nicrophorus* develop in the presence of a highly valuable and decaying resource; individuals need to hatch, feed and disperse before the carcass is either claimed by another animal or becomes unsuitable for development. This strong selection for fast development might be reflected by a trade-off between a well-protected but slow developing egg and a fast-developing but less protected egg. Similar trade-offs between growth and immune competence are known from plants [49], birds [50] and insects [51,52]. Although additional experiments are needed to confirm the relation between rapid development and the lack of immune competence in insect eggs, the high survival and poor immune competence of both *N. vespilloides* and *D. melanogaster* eggs under normal conditions suggests that fast development is obtained at the expense of immune competence.

## Conclusions

Our work builds upon previous studies demonstrating the profound costs to *N. vespilloides* from rearing their offspring in the presence of microbial competitors or pathogens in the soil environment. Although parental care in this species can serve to mitigate some of these risks, our data suggest that at least direct care does not extend to eggs. The indirect effects of fumigation with volatiles of the surrounding microhabitat might be important, however this conjecture requires further testing. The lack of direct parental provisioning of eggs may result from a trade-off between egg protection and carcass maintenance. Similarly, the lack of immune competence may be caused by a trade-off between immunity and the need for rapid growth on a rich and ephemeral resource. Although similar life-history trade-offs are known in a broad range of species, we are unaware of results showing this trade-off for eggs. This result therefore has broad implications owing to the obvious importance of egg survival for lifetime reproductive success, and suggests the need to investigate the development of immune competence more broadly as a function of developmental timing.

### Data accessibility

All data used in this manuscript are present in the manuscript and its supporting information.

## Additional file

Additional file 1: Figure S1. Expression differences between infected eggs and infected larvae. Expression of antimicrobial peptides in general is significantly higher in larvae than in eggs. Only the difference in Defensin 2 was small, which is expected as it is the least induced antimicrobial peptide in this study.
