# Supplementary figures and images for: Egg survival is reduced by grave-soil microbes in the carrion beetle, Nicrophorus vespilloides
Source: BMC Evol Biol. 2014 Sep 27;14:208. doi: 10.1186/s12862-014-0208-x (PMC4189599; doi:10.1186/s12862-014-0208-x)

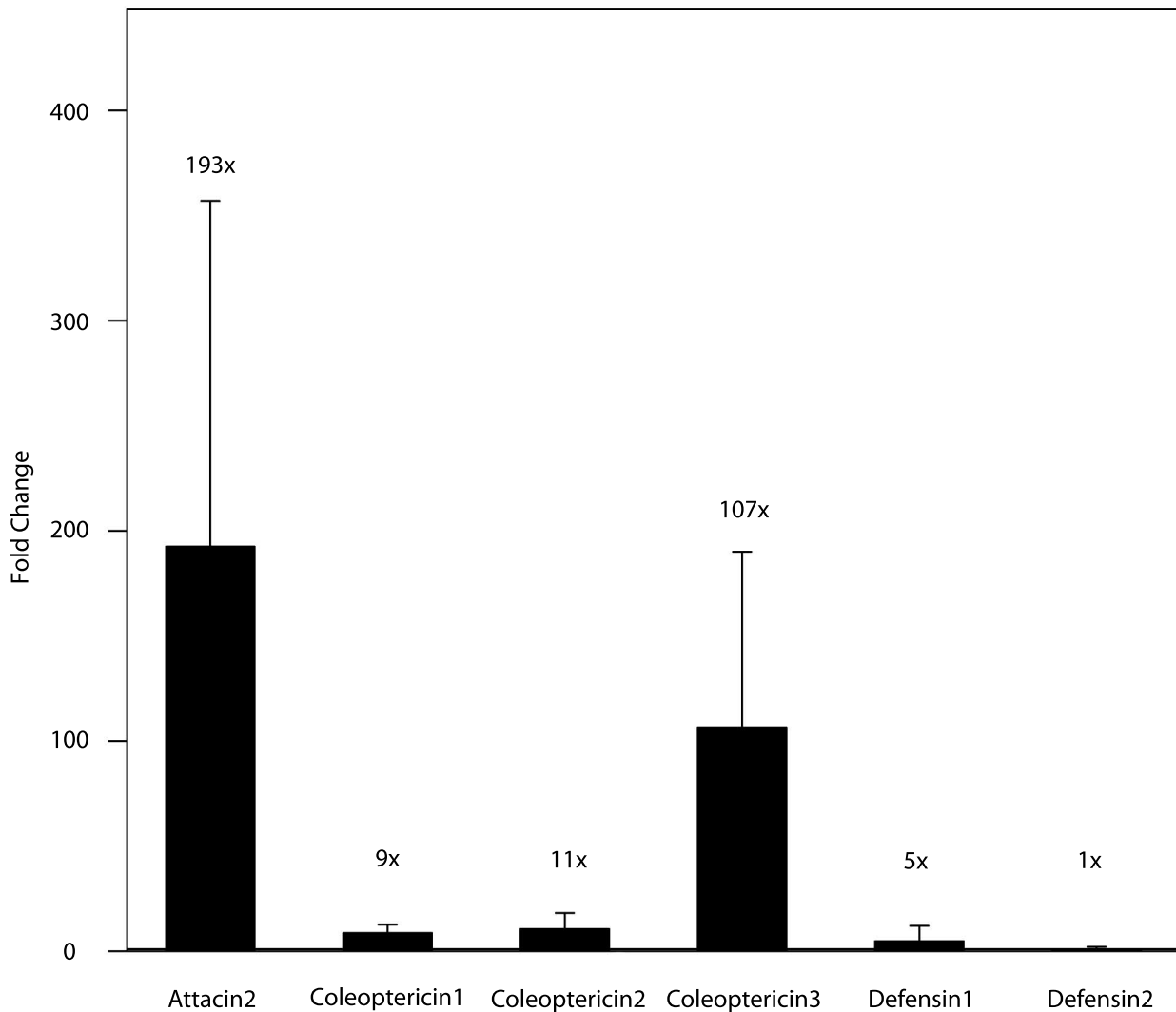

Supplement: Additional file 1: Figure S1. — Expression differences between infected eggs and infected larvae. Expression of antimicrobial peptides in general is significantly higher in larvae than in eggs. Only the difference in Defensin 2 was small, which is expected as it is the least induced antimicrobial peptide in this study. [file 12862_2014_208_MOESM1_ESM.pdf]
